# Proteolytic remodeling by Yme1 enables mitochondrial-derived compartment formation

**DOI:** 10.64898/2026.04.03.716366

**Published:** 2026-04-05

**Authors:** Sai Sangeetha Balasubramaniam, Amy E. Curtis, Jonathan R. Friedman, Adam L. Hughes

## Abstract

Mitochondrial-derived compartments (MDCs) are remodeling domains that form from the outer mitochondrial membrane during metabolic and proteotoxic stress and selectively sequester hydrophobic membrane proteins. Although MDC formation depends on mitochondrial lipid composition and occurs at organelle contact sites, the molecular mechanisms that permit their biogenesis remain poorly defined. Here we identify the conserved inner mitochondrial membrane i-AAA protease Yme1 as a critical regulator of MDC formation. Loss of Yme1 blocks MDC biogenesis in response to multiple stressors, and this requirement depends on its proteolytic activity rather than secondary defects in mitochondrial morphology. Quantitative mitochondrial proteomics under MDC-inducing conditions revealed Yme1-dependent remodeling of lipid transfer proteins of the Ups family and components of the MICOS complex. Disruption of either pathway partially restores MDC formation in *yme1Δ* cells, while combined perturbation substantially bypasses the requirement for Yme1. Finally, Yme1 overexpression drives MDC formation in the absence of stress, although this activity remains constrained by metabolic conditions. Together, these findings support a model in which Yme1-dependent proteolysis relieves lipid- and MICOS-dependent constraints to permit MDC formation.

## Full Text Availability

The license terms selected by the author(s) for this preprint version do not permit archiving in PMC. The full text is available from the preprint server.

